# Brainstem facilitations and descending serotonergic controls contribute to visceral nociception but not pregabalin analgesia in rats

**DOI:** 10.1016/j.neulet.2012.05.009

**Published:** 2012-06-21

**Authors:** Shafaq Sikandar, Kirsty Bannister, Anthony H. Dickenson

**Affiliations:** Department of Neuroscience, Physiology and Pharmacology, University College London, WC1E 6BT London, UK

**Keywords:** Visceral nociception, Rostral ventromedial medulla (RVM), Visceromotor response (VMR), 5-HT, Pregabalin

## Abstract

Pro-nociceptive ON-cells in the rostral ventromedial medulla (RVM) facilitate nociceptive processing and contribute to descending serotonergic controls. We use RVM injections of neurotoxic dermorphin-saporin (Derm-SAP) in rats to evaluate the role of putative ON-cells, or μ-opioid receptor-expressing (MOR) neurones, in visceral pain processing. Our immunohistochemistry shows that intra-RVM Derm-SAP locally ablates a substantial proportion of MOR and serotonergic cells. Given the co-localization of these neuronal markers, some RVM ON-cells are serotonergic. We measure visceromotor responses in the colorectal distension (CRD) model in control and Derm-SAP rats, and using the 5-HT_3_ receptor antagonist ondansetron, we demonstrate pro-nociceptive serotonergic modulation of visceral nociception and a facilitatory drive from RVM MOR cells. The α_2_δ calcium channel ligand pregabalin produces state-dependent analgesia in neuropathy and osteoarthritis models relating to injury-specific interactions with serotonergic facilitations from RVM MOR cells. Although RVM MOR cells mediate noxious mechanical visceral input, we show that their presence is not a permissive factor for pregabalin analgesia in acute visceral pain.

## Highlights

► Descending 5-HT_3_ receptor-mediated controls are pro-nociceptive in visceral pain. ► Some μ-opioid receptor-expressing RVM cells, putative ON-cells, are serotonergic. ► Putative RVM ON-cells mediate basal visceral pain responses in the CRD model. ► Pregabalin inhibits evoked visceral pain in rats with ablated RVM ON-cells. ► State-dependent pregabalin analgesia in neuropathy does not apply to visceral pain.

## 1 Introduction

The rostral ventromedial medulla (RVM) of the caudal brain stem engages descending facilitatory and inhibitory pathways to alter spinal nociceptive processing [28]. Brainstem modulation of visceral pain responses in the colorectal distension (CRD) model of acute visceral pain is well established and RVM descending projections provide a primary source of spinal serotonin (5-HT) [5,10].

Several studies demonstrate serotonergic facilitatory modulation onto the spinal cord through 5-HT_3_ receptors in neuropathic [17], inflammatory [16] and visceral pain models [22]. The 5-HT_3_ receptor antagonist ondansetron reduces the excitability of dorsal horn neurones [7]. Here, we pharmacologically investigate the role of brainstem descending serotonergic modulation in visceral pain using the CRD model and ondansetron.

RVM neurones are classified ON, OFF and NEUTRAL based upon firing patterns following noxious somatic stimulation; ON-cells increase firing immediately before a nocifensive response [9]. However, RVM neurones do not always respond in the same direction to visceral and cutaneous stimulation [5,19]. RVM ON-cells are directly hyperpolarised by μ-opioids, thus referred to as MOR cells here [9]. Opioidergic sensitivity and the neurotoxin saporin allows for site-specific ablation of putative RVM ON-cells [15]. Following conjugation with the MOR agonist dermorphin, GPCR-internalization of saporin causes apoptotic cell death, whereas saporin alone has limited intracellular access and thus minimal toxicity [4]. Furthermore, a significant proportion of spinally-projecting μ-opioid-responsive neurones are serotonergic [11,20,24], and so it is likely that some ON-cells are serotonergic.

Targeted Derm-SAP ablation has previously demonstrated a pro-nociceptive role of RVM MOR cells in neuropathy and pancreatitis [15,22,23] and a key permissive role of these cells and 5-HT_3_ receptor-mediated descending facilitations in the analgesic efficacy of pregabalin using somatic stimuli [3]. A recent study reports subcortical sites of pregabalin action in visceral pain [13]. We use intra-RVM Derm-SAP to evaluate the role of putative RVM ON-cells in visceral pain responses and whether these cells also exert a permissive action on pregabalin analgesia in visceral pain. Thus, this study provides an anatomical and pharmacological substrate for exploring modulation of visceral pain responses mediated by the brainstem.

## 2 Materials and methods

All studies used male Sprague Dawley rats supplied by Biological Services (University College London, UK). All procedures were approved by Home Office (UK) and in agreement with IASP guidelines [29].

### 2.1 Electromyography recordings

Rats (250–300 g) were maintained on anaesthesia at 1% (v/v) isoflourane in a mixture of N_2_O (66%, v/v) and 0_2_ (33%) through a tracheal cannula. An electromyography (EMG) electrode was sown into the right external oblique muscle and rats were secured to a stereotaxic frame with a heating blanket to maintain a core temperature of 37 °C. CRDs were produced by manually inflating a colorectal balloon and a pressure amplifier measured degree of inflation in mmHg [19]. The electrode was connected to a Neurolog system with a 1401 interface and Spike2 software (Cambridge Electronic Design, UK). Filtered signals from muscle activity were integrated (NL703 Module, Digitimer Limited, UK) and measured as visceromotor responses (VMR).

The protocol for CRD administration was carried out as previously described [19]. 30 mmHg was considered the cut-off between low- and high-threshold stimulation [12]. Once control EMG values were obtained for distensions from 10 mmHg to 80 mmHg (integrated EMG values did not differ more than 15% from each other), a drug was administered (pregabalin, 30 mg/kg s.c.; ondansetron, 50 μg in 25 μl i.t. injected into L6-S1 intervertabrae space). Series of distensions were performed and VMR recorded 20 and 60 min after drug administration.

To produce visceral hyperalgesia, mustard oil (0.25% in 1 ml mineral oil) was applied intra-anally in the area occupied by the CRD balloon, after control distensions. Thirty minutes were allowed for hyperalgesia to develop. Drug was administered 10 min after mustard oil (MO) so VMR was recorded 30 and 70 min after mustard oil application (corresponding to 20 and 60 min after drug administration).

### 2.2 Intra-RVM injections

Rats (130 g) were anaesthetized with ketamine hydrochloride (60 mg/kg i.p.) and medetomidine hydrochloride (0.5 mg/kg i.p.). Animals were secured to a stereotaxic frame and saporin or cytotoxic dermorphin-saporin (3 pmol in 1 μl; Advanced Targeting Systems, San Diego, CA) was injected into the RVM (0.0 mm mediolateral, 8.5–9.5 mm caudal from bregma, 9.0 mm dorsal from the dura matter). Atipamezole hydrochloride (1 mg/kg s.c.) was used to reverse anaesthesia. Rats were housed in cages under a 12-h alternating light/dark cycle with ad libitum access to food and water. 28 days were allowed for full cytotoxicity [15] before use in immunohistochemistry and EMG recordings.

### 2.3 Immunohistochemistry

Rats were anaesthetized with pentobarbitone sodium (200 mg i.p.; Merial Animal Health Ltd., UK) and transcardially perfused with 300 ml saline (0.9%, w/v) with heparin (5000 IU/l saline; LEO Laboratories, UK) and 300 ml of 4% paraformaldehyde (VWR, UK) in 0.6 M phosphate buffer solution (PBS). The spinal cord and hindbrain were removed. Tissue was post-fixed and stored in a cryoprotectant solution of 30% (w/v) sucrose in 0.1 M PBS and 0.01% (w/v) sodium azide (Sigma Aldrich, UK) for 3 days. RVM tissue was collected serially in 40 μm sections.

RVM sections were double-labelled for tryptophan hydroxylase (TPH, the catalyst for serotonin synthesis as a marker of 5-HT) and MOR. For MOR staining, a rabbit polycolonal μ-opioid receptor primary antibody (1:10,000 TTBS; Neuromics, MN, USA, RA10104) was used with tyramide signal amplification (TSA, Perkin Elmer, USA) and the avidin biotin labelled complex (ABC Elite, Vector Laboratories), followed by incubation with fluorescein isothiocyanate (FITC, 1.67:1000 TTBS). Sections were then incubated with mouse monoclonal anti-TPH primary antibody (1:1000 TTBS; Sigma–Aldrich, UK, T0678) and fluorescent Alexa Red fluorescent (Alexa Red 594, 1:500 TTBS; Invitrogen, UK). Every third RVM section with on-target injection sites was analysed using a fluorescence microscope at 10× magnification with appropriate wavelengths. Regions for immunopositive cell analysis were delineated with reference to the rat brain atlas and restricted to 10.5–11.5 mm caudal from bregma [14].

### 2.4 Statistical analysis

Integrated EMG values with subtracted baselines were normalized for each animal to mean controls EMG. Normalized EMG data was evaluated for normal distribution and eligibility for ANOVA analysis with both the Kolmogorov–Smirnov (with Dallal–Wilkinson–Lilliefor *P* value) and the D’Agostino and Pearson omnibus normality tests. A two-way ANOVA with repeated measures and Bonferroni post-tests was used to determine significant changes in VMR throughout the range of CRD pressures between drug time-points. A one-way ANOVA with Bonferroni post-tests was used for area-under-curve comparisons.

Immunohistochemistry analysis was blinded using tissue from 4 rats per group (naïve, SAP and Derm-SAP) and 3 sections per animal. Templates for regions for immunopositive cell analysis were delineated for quantifying total number MOR and TPH immunopositive cells. A one-way ANOVA with Bonferroni post-tests was used to determine the difference between RVM cell counts of naïve, SAP and Derm-SAP rats.

Statistical significance for all analysis was set at (*) *P* < 0.05, (**) *P* < 0.01 and (***) *P* < 0.001 and data presented as mean ± SEM.

## 3 Results

### 3.1 Serotonergic modulation of visceromotor responses

**Fig. 1:**
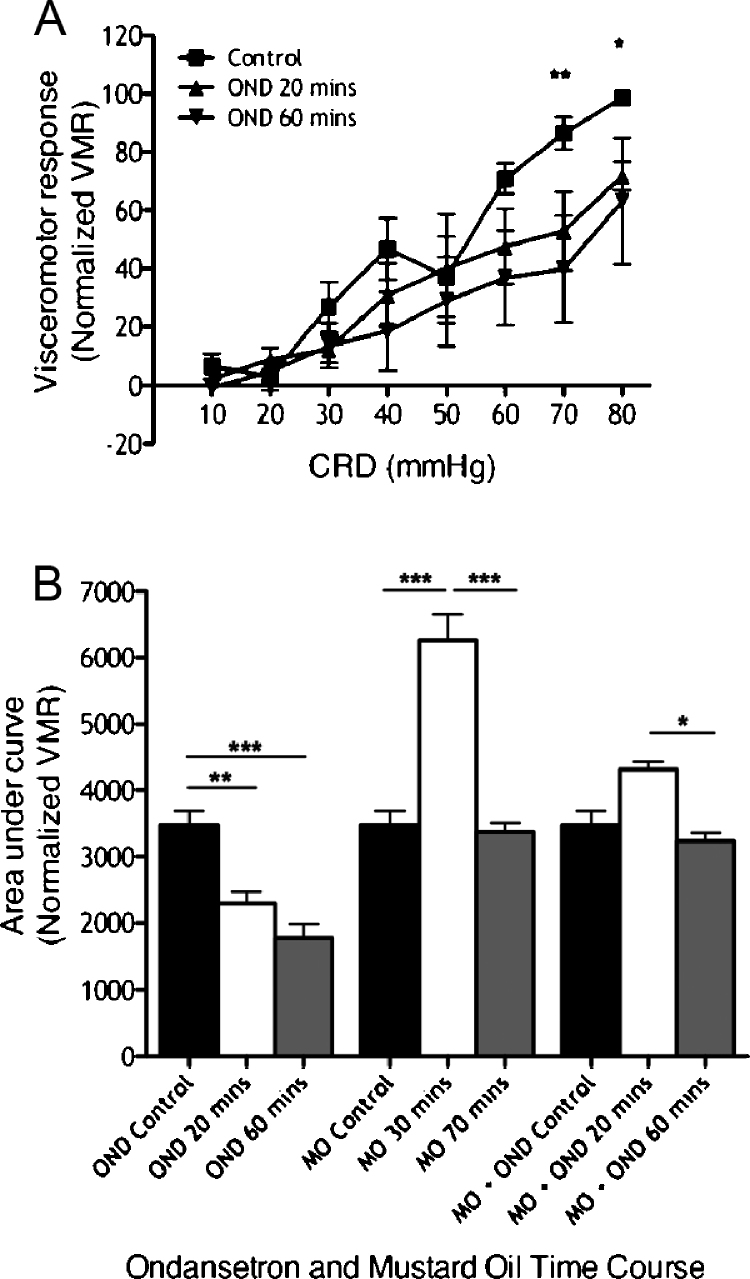
Intrathecal ondansetron reduces VMR evoked by noxious CRD and prevents development of visceral hyperalgesia induced by intracolonic mustard oil. (A) VMR evoked by a range of innocuous to noxious CRD pressures before and after ondansetron administration (*n* = 6; *P < 0.05, **P < 0.01 control vs. 60 min). (B) Collective area-under-curve data of rats given ondansetron (OND; *n* = 6), intracolonic mustard oil (MO; *n* = 8), and intracolonic mustard oil and ondansetron (MO + OND; *n* = 6) (*P < 0.05, **P < 0.01, ***P < 0.001).

Control visceromotor responses (VMR) prior to drug administration were clearly graded with increasing CRD pressures from 10 mmHg to 80 mmHg. Effects of spinal ondansetron, a 5-HT_3_ antagonist, were measured at 20 and 60 min. Ondansetron reduced VMR at noxious CRD pressures (70–80 mmHg), revealing a role for 5-HT_3_ receptor-mediated facilitatory modulation of nociceptive VMR (Fig. 1A; time variable *F*[2,80] = 11.93, *P* < 0.0001; pressure variable *F*[7,80] = 10.28, *P* < 0.0001). Overall VMR comparisons illustrate rapid onset of ondansetron inhibition 20 min after its administration (Fig. 1B first three bars; *F*[2,15] = 18.48, *P* < 0.0001).

Intracolonic mustard oil produces a transient visceral hyperalgesia where VMR are significantly potentiated in a time-dependent manner, peaking at 30 min after mustard oil application, with recovery of VMR to control responses 70 min after mustard oil application (Fig. 1B middle three bars; *F*[2,15] = 36.75, *P* < 0.0001). Ondansetron blocks this VMR potentiation (last three bars; *F*[2,15] = 13.09, *P* < 0.0001).

### 3.2 Cytotoxic and visceromotor effects of intra-RVM Derm-SAP injections

**Fig. 2:**
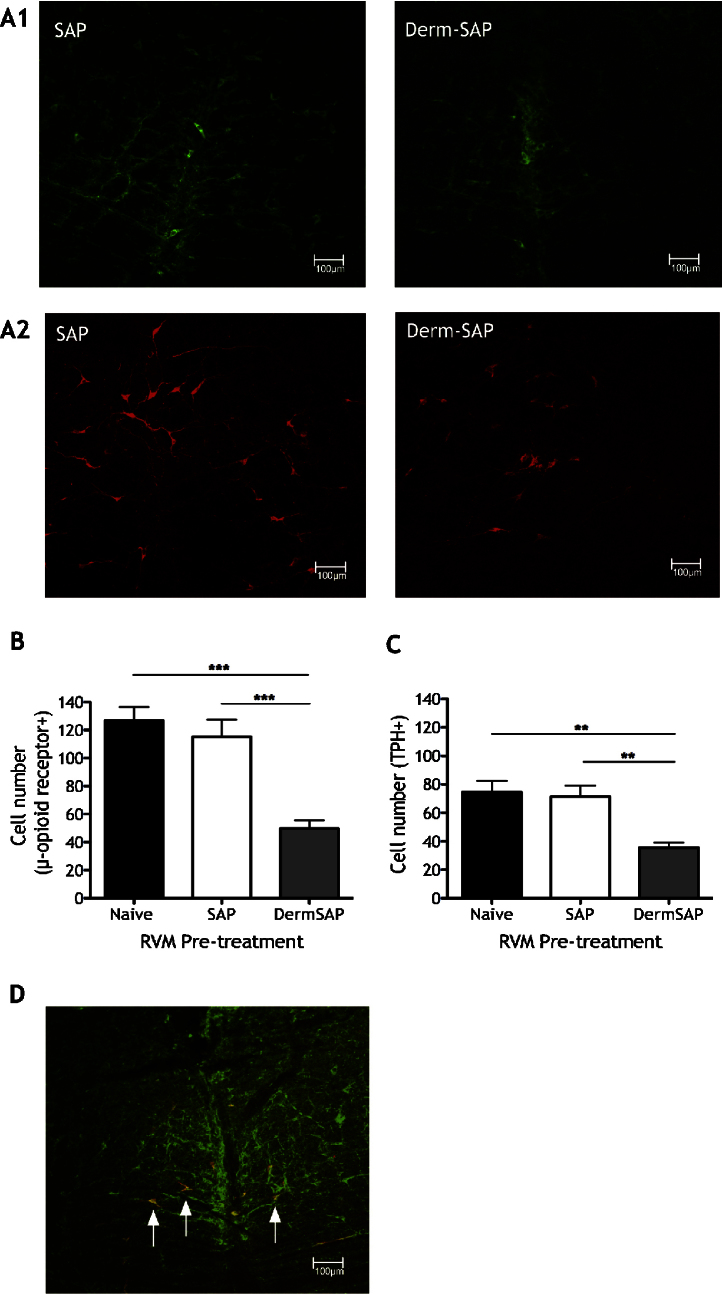
Reduction of RVM MOR and TPH immunoreactivity in Derm-SAP rats. (A) Photomicrographs of representative RVM sections from SAP and Derm-SAP rats for MOR immunoreactivity (A1; labelled neurones in green) and TPH immunoreactivity (A2; labelled neurones in red). (B) MOR-expressing cell count in RVM sections from naïve, SAP and Derm-SAP rats (***P < 0.001). (C) TPH-positive cell count in RVM sections from naïve, SAP and Derm-SAP rats (**P < 0.01). (D) Confocal photomicrograph of an RVM section showing co-localization for TPH (red) and MOR (green) immunoreactivity (some double-labelled neurones indicated by arrows).

Derm-SAP treatment significantly reduces RVM MOR cell counts compared to control SAP or naïve rats (Fig. 2A and B; *F*[2,9] = 19, *P* < 0.0001). Some RVM MOR cells also express TPH immunoreactivity, a marker for 5-HT (Fig. 2D). Thus, Derm-SAP treatment also significantly reduces TPH+ RVM cell counts (Fig. 2A and C; *F*[2,9] = 11.01, *P* < 0.0001). Importantly, there is no significant difference in MOR or TPH expression between naïve and SAP groups.

**Fig. 3:**
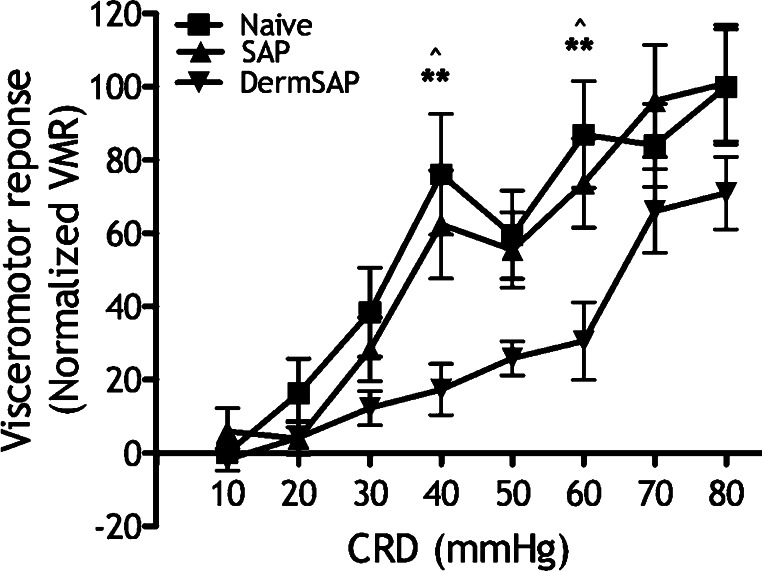
Reduction of basal VMR in Derm-SAP rats. Control VMR of naïve (*n* = 6), SAP (*n* = 8) and Derm-SAP rats (*n* = 8; **P < 0.01 naïve vs. Derm-SAP, ^^^*P* < 0.05 SAP vs. Derm-SAP).

**Fig. 4:**
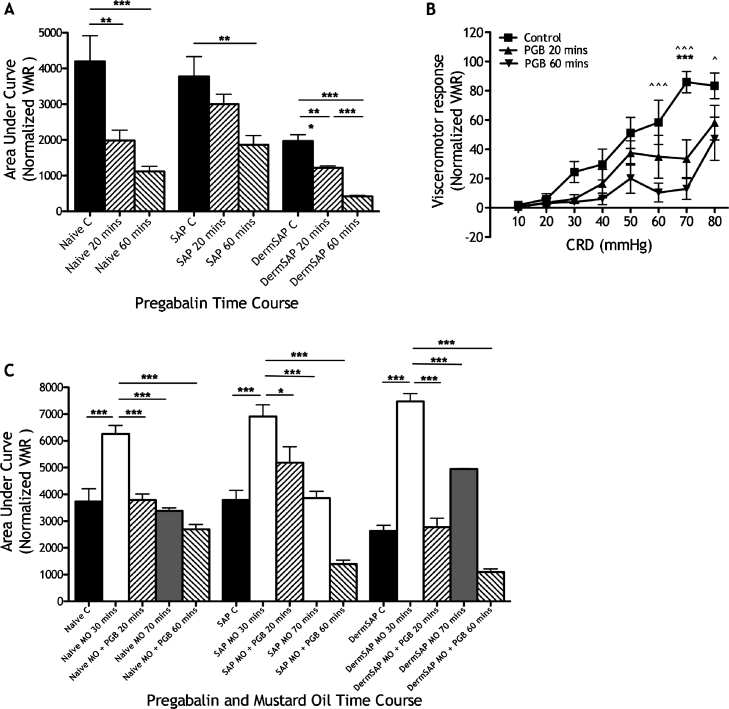
Pregabalin analgesia in Derm-SAP rats. (A) Overall VMR following systemic pregabalin in naïve (*n* = 6), SAP (*n* = 6) and Derm-SAP rats (*n* = 6) with controls normalized to the control VMR of naïve rats (*P < 0.05, **P < 0.01, ***P < 0.001). Note the significant reduction of control VMR in Derm-SAP rats compared to naïve and SAP rats. (B) VMR evoked by a range of innocuous to noxious CRD pressures before and after pregabalin administration in Derm-SAP rats (*n* = 6; ***P < 0.001 control vs. 20 min, ^^^*P* < 0.05, ^^^^*P* < 0.01, ^^^^^*P* < 0.001 control vs. 60 min). (C) Collective data of naïve, SAP and Derm-SAP rats with intracolonic mustard oil (naïve MO, *n* = 8; SAP MO, *n* = 8; Derm-SAP MO, *n* = 8) and naïve, SAP and Derm-SAP rats with pregabalin and intracolonic mustard oil (naïve MO + PGB, *n* = 7; SAP MO + PGB, *n* = 8; Derm-SAP MO + PGB, *n* = 8) – all with VMR normalized to the controls of naïve rats. Significant differences are only shown relative to 30 min after mustard oil application to illustrate pregabalin inhibition of visceral hyperalgesia (*P < 0.05, ***P < 0.001).

VMR of Derm-SAP rats is graded with increasing CRD pressures, but lower than overall VMR of naïve and SAP rats evoked at noxious pressures, significantly at 40 and 60 mmHg (Fig. 3; time variable *F*[2,80] = 11.93, *P* < 0.0001; pressure variable *F*[7,80] = 10.28, *P* < 0.0001). Also note the lower control VMR of Derm-SAP rats (Fig. 4A controls; *F*[2,11] = 6.80, *P* < 0.01).

### 3.3 Pregabalin modulation of visceromotor responses in acute pain and visceral hyperalgesia following Derm-SAP treatment

Systemic pregabalin (30 mg/kg s.c.) inhibits VMR consistently in naïve rats (*F*[2,11] = 12.25, *P* < 0.0001), SAP rats (*F*[2,11] = 6.081, *P* < 0.001) and Derm-SAP rats (*F*[2,11] = 50.83, *P* < 0.0001), irrespective of the basal levels of activity (Fig. 4A). Systemic pregabalin inhibits VMR evoked by noxious CRD pressures in Derm-SAP rats, significantly at 60–80 mmHg CRD (Fig. 4B; time variable *F*[2,80] = 26.42, *P* < 0.0001; pressure variable *F*[7,80] = 15.51, *P* < 0.0001).

Visceral hyperalgesia is produced by intracolonic mustard oil in all groups, which is blocked by systemic pregabalin (Fig. 4C; Derm-SAP rats (*F*[4,3] = 16.96, *P* < 0.0001); naïve rats (*F*[4,3] = 24.86, *P* < 0.0001); SAP rats (*F*[4,3] = 9.731, *P* < 0.01)). Pregabalin continues to inhibit VMR 60 min after administration, corresponding to 70 min after mustard oil application (when VMR regains control levels in rats only treated with mustard oil and no modulatory drug).

## 4 Discussion

We used a receptor-specific antagonist to demonstrate a pro-nociceptive role of spinal 5-HT_3_ receptors in the CRD model of acute visceral pain. The Derm-SAP ablation technique revealed a role of RVM ON-cells (MOR neurones) in regulating visceral pain processing, but not the analgesic efficacy of pregabalin. This study highlights the role of brainstem modulation of visceral pain responses and reveals a lack of state-dependence for pregabalin analgesia in acute visceral pain.

### 4.1 Intra-RVM Derm-SAP ablations include serotonergic ON-cells

We established that intra-RVM Derm-SAP effectively ablates a substantial proportion of MOR cells and a proportion of serotonergic cells in the RVM. The parallel reduction in labelling for these markers and their co-localization suggests that *some* MOR RVM cells are serotonergic. Other reports of significant proportions of spinally-projecting μ-opioidergic RVM neurones with serotonergic immunoreactivity support our findings [11,20,24]. Indeed, we refer to MOR cells as putative ON-cells by definition of μ-receptor expression, not neuronal firing [9]. The idea that ON-cells are serotonergic is conflicted by some reports suggesting that serotonergic neurones are contained as one physiological class in the RVM [6]. However, these reports do not confirm that ON-cells are *not* serotonergic and discrepancies may also be attributed to a small number of cells sampled, which is ultimately not representative of the heterogeneous RVM population [11,27].

### 4.2 RVM ON-cells and serotonergic descending facilitations in visceral pain

Robust inhibitory effects of ondansetron in our study clearly argue for pro-nociceptive serotonergic modulation of visceral pain. The majority of spinal 5-HT arises from descending RVM projections [10] – a pro-nociceptive system in neuropathy and inflammation [25]. We have shown that a proportion of RVM MOR cells are serotonergic, and further demonstrated a facilitatory role of these cells in acute visceral nociception, given that intra-RVM Derm-SAP inhibits full expression of basal visceral pain responses.

Putative RVM ON-cells are thought to infer selectivity in modulating spinal sensory transmission of noxious stimuli without affecting normal central processing of low-intensity input, although this may change after neuropathy [3]. This is likely related to the preferential expression of 5-HT_3_ receptors on nerve terminals of small-diameter primary afferent fibres [26]. Indeed, we show that 5-HT_3_ receptor blockade selectively reduces VMR evoked by noxious CRD pressures. We also find that intra-RVM Derm-SAP produces lower basal VMR, significantly reduced in the noxious range of CRD pressures compared to control rats. Inactivating the RVM with lignocaine or transecting the spinal cord also selectively reduces mechanical hypersensitivities in neuropathic animals [3,21]. These findings reinforce a sub-modality specificity of brainstem regulation and response to noxious mechanical stimuli.

We use intracolonic mustard oil as a model of transient visceral hyperalgesia. In Derm-SAP rats pre-treated with intracolonic mustard oil, a transient enhancement of VMR develops similar to control rats. Although we observe a partial loss in coding of basal VMR in Derm-SAP rats, this reduction in facilitatory drive from RVM ON-cells following Derm-SAP treatment is not sufficient to attenuate the development of central hypersensitivity during colonic inflammation. Similarly, Derm-SAP rats show normal hypersensitivity in early stages of chronic pain development [23]. Thus RVM MOR cells provide descending pro-nociceptive controls on acute visceral pain responses without significantly affecting the development of hypersensitivities in acute inflammation. Sufficient facilitatory drive must be retained in Derm-SAP rats – from spared RVM MOR cells or from other central neurones – to contribute to VMR evoked by noxious CRD and contribute to potentiated VMR in our acute visceral hyperalgesia model. An added complication is the potential facilitatory role of other RVM neurones (OFF- and NEUTRAL-cells) in visceral processing [5,19].

### 4.3 Pregabalin analgesia in acute visceral pain vs. state-dependent actions of pregabalin in neuropathy

Pregabalin binds with the ubiquitous α_2_δ subunit of voltage-gated calcium channels to impair calcium channel trafficking [2]. Its analgesic actions in neuropathy and osteoarthritis are thought to rely on permissive factors – these include a pathological state and injury-specific interactions with descending 5-HT_3_-receptor mediated facilitations form the brainstem [3,16]. Indeed, intra-RVM Derm-SAP has previously demonstrated the permissive role of these ON-cells in pregabalin analgesia in neuropathy [3]. On the other hand, pregabalin analgesia has recently been demonstrated in a model of opioid-induced hyperalgesia where pathophysiology is lacking [1], and other studies report analgesic effects of pregabalin in acute visceral pain [18,19]. We also show here that systemic pregabalin significantly reduces evoked visceral pain in Derm-SAP rats in normal conditions and during transient visceral hyperalgesia, thereby dismissing any permissive role of putative RVM ON-cells in the analgesic efficacy of pregabalin in acute visceral pain (unlike neuropathy and osteoarthritis conditions). As discussed above, we cannot exclude the possibility of actions of pregabalin on other RVM neurones or locus coeruleus neurones in producing analgesia [8].

We have recently demonstrated an activity-dependent role of RVM neurones in VMR and a sensitivity of RVM ON-cells to pregabalin [19]. Here, the lack of effect on acute visceral pain responses and development of visceral hyperalgesia following ablation of RVM MOR cells could be explained by the lack of correspondence in RVM cell activity to visceral and somatic stimulation [5,19]. Accordingly, all three RVM cell types can behave as ON-cells when visceral stimuli are used to provide a permissive facilitatory drive that allows pregabalin actions. Similar explanations could underlie the resistance of mustard oil-induced hyperalgesia to the ablative technique.

## 5 Conclusions

We show that 5-HT_3_ receptor-mediated descending facilitation modulates acute visceral pain. We also use Derm-SAP ablation of putative RVM ON-cells to reveal their role in regulating basal visceral pain responses. Nevertheless, descending facilitations mediated by these cells is not necessary for the development of visceral hyperalgesia or for the analgesic efficacy of pregabalin. The latter contradicts findings in some somatic pain models, so the mechanisms of action of pregabalin must differ between somatic and visceral pain states. However, a shared interaction may be the shift in the balance of descending modulation from the brainstem, where enhanced descending facilitatory controls (or a reduction in inhibitory influences) produced by acute visceral stimuli produce a permissive physiological state of central excitability for pregabalin to exert inhibitory actions.
